# How new is the new philosophy of psychiatry?

**DOI:** 10.1186/1747-5341-2-22

**Published:** 2007-10-20

**Authors:** Damiaan Denys

**Affiliations:** 1 Department of Psychiatry, AMC, University of Amsterdam, PA.2-179, PO Box 75867, 1070 AW Amsterdam, The Netherlands

## Abstract

In their recent paper, Natalie Banner and Tim Thornton evaluate seven volumes of the Oxford University Press series “International Perspectives in Philosophy and Psychiatry,” an international book series begun in 2003 focusing on the emerging interdisciplinary field at the interface of philosophy and psychiatry. According to Natalie Banner and Tim Thornton, the series represents a clear indication that the interdisciplinary field of philosophy of psychiatry has been flourishing lately. Philosophers and psychiatrists face a “new philosophy of psychiatry”. However, the optimism which the “new” philosophy of psychiatry celebrates is precisely the exiling of philosophy from the foundations of psychiatry. The 150 year old belief that psychopathology cannot do without philosophical reflection has virtually disappeared from common psychiatric education and daily clinical practice. Though the discipline of psychiatry is particularly suited to contributions from philosophy, the impact of philosophy on psychiatry nowadays remains limited. With some exceptions, philosophical papers are embedded in a philosophical context inscrutable to ordinary psychiatrists. Much current philosophical work is perceived by psychiatrists as negativistic. I would encourage the field of psychiatry to incorporate once again basic philosophical attitudes which render possible true dialogue with philosophy and enrich both disciplines. The views developed here should not discredit the value and importance of Natalie Banner and Tim Thornton’s paper and the excellent series “International Perspectives in Philosophy and Psychiatry.” As Jaspers said “Everybody inclined to disregard philosophy will be overwhelmed by philosophy in an unperceived way”.

## 

In their recent paper, Natalie Banner and Tim Thornton evaluate seven volumes of the Oxford University Press series "International Perspectives in Philosophy and Psychiatry". Launched in 2003, "International Perspectives in Philosophy and Psychiatry" is an international book series focusing on the emerging interdisciplinary field at the interface of philosophy and psychiatry.

Natalie Banner and Tim Thornton identify three broad interconnected themes in the series: the role of values in psychiatric diagnosis and treatment; the question of the place of understanding subjects' experiences, their meanings and the relationship of understanding to natural scientific explanation, and the scientific status of the 'facts' or 'evidence' that contribute towards psychiatric diagnoses. The three themes correspond with the three main parts of Tim Thornton's new book "Essential Philosophy of Psychiatry" meant to be a concise introduction to the growing field of philosophy of psychiatry. The first part, *Values*, outlines the debate about whether diagnosis of mental illness is essentially value-laden and argues that the prospects for reducing illness or disease to plainly factual matters are poor. The second part, *Meanings*, examines the central role of understanding and a shared first person perspective, both against attempts to reduce meaning to basic information-processing mechanisms and to explain away the difficulties of understanding psychopathology. The third part, *Facts*, shows the importance of uncodified clinical judgments, both in assessing the validity of psychiatric taxonomy and in the application of Evidence Based Medicine.

According to Natalie Banner and Tim Thornton, the series represents a clear indication that the interdisciplinary field of philosophy of psychiatry has been flourishing lately. There has been recent growth in the philosophy of psychiatry during the past fifteen years. Philosophers and psychiatrists face a "new philosophy of psychiatry" in addition to analytic philosophy and to the broader interpretation of mental health care.

How new is this new philosophy of psychiatry? Does the new philosophy really impact on the field of psychiatry? Should we share Natalie Banner and Tim Thornton's optimism?

Since psychiatry has been established as a field of medicine, psychiatric literature has always been full of philosophical thought and direct reference to philosophy. "*Just meditations for the philosopher who, liberated from the daily turmoil, walks through a psychiatric hospital! He will find the same ideas, the same errors, the same passions, the same ill-fated: it's the same world, but in this house, traits are more pronounced, nuances much sharper, colors more vivid, lives more shattered, because man are naked, they don't conceal their thoughts, hide their shortcomings, they don't draw on their passions to articulate charming seduction, on their vices to express deceiving appearances" *[1]. The broad themes: values, meanings and facts identified by Natalie Banner and Tim Thornton in the recent series have been examined in psychiatry for many years. Bertrand Morel discusses in his "Traité des maladies mentales" (1850) the role of political and religious values in psychiatry referring to Rousseau and Locke [2]. Jaspers' project of the General Psychopathology (1913) originally aimed at examining facts and perspectives in psychiatry thereby using "meaning" from Dilthey (1900) as a methodological tool [3]. The validity of psychiatric diagnoses, the relation between scientific explanation and human understanding, and the scientific status of psychiatric facts have been studied extensively by Continental phenomenological psychiatrists. For my part, the recent themes of the new philosophy of psychiatry are just an extension or repetition of earlier work of the last centuries. There has always been a longstanding debate on truth, method and the scientific status of psychiatric knowledge, and questions about the possibility of true knowledge in psychiatry are inherent to psychiatric thinking.

Nevertheless, I agree with Natalie Banner and Tim Thornton that something has radically changed within the field of psychiatry and philosophy during the past fifteen years. The novelty is not that philosophy has reconquered psychiatry, but that psychiatry has lost philosophy. Philosophical thinking used to be embedded in psychiatry. This was self-evident since psychiatry and philosophy share interest in the same matters – reality, freedom, personal identity, social reality, perception, free will, thought and affect. However, the belief that psychopathology cannot do without philosophical reflection, so obvious the last 150 years, has recently vanished. Reflecting, conceptual thinking, questioning, and criticizing have all virtually disappeared from common psychiatric education and daily clinical practice. Jaspers was a resident in psychiatry and not a philosopher when he wrote his "General Psychopathology." Unfortunately, the vital, basic philosophical attitude of naive astonishment towards psychiatric phenomena is no longer part of residency training. Philosophy has left the psychiatric building. It is exiled from psychiatry, externalized and sequestered in the "new" philosophy of psychiatry.

The loss of critical philosophical thinking in psychiatry has led on one hand to the shameful conclusion that zoologists are much more accurate and subtle than psychiatrists in the observation of behavior. "The available analysis of the phenomenology of compulsive rituals pales before elegant observations of analogous behaviors in fish and birds" Thomas Insel (1988) [4]. The loss has led on the other hand to the necessity of two different disciplines both struggling to detect a fruitful crossover. I agree with Natalie Banner and Tim Thornton that the discipline of psychiatry is particularly suited to contributions from philosophy. However, the impact of philosophy on psychiatry is still limited. Though the conditions for systematic thought over the last decades have changed fundamentally – not only phenomenology is at our disposal but the philosophy of mind – they have not yet been used widely to deal with psychopathological problems. The focus in training is on scientific knowledge, such as clinical neuroscience, behavioural and social sciences. There is very little content devoted to anthropology and philosophy in relation to psychiatry [5]. The language of academic philosophy is not the language of bedside psychiatry. With some exceptions, philosophical papers are so dense, so laden with jargon, and so embedded in a philosophical context inscrutable to the ordinary psychiatrist that their message is lost. Moreover, much of current philosophical work is criticism, emphasizing the limitations of modernist thinking and rejecting its claims, and critically analyzing the conceptual foundations of academic psychiatry. The majority of the reviewed seven volumes of the Oxford University Press series "International Perspectives in Philosophy and Psychiatry" criticize present psychiatric values, meanings and facts. Coming from outside, the criticism is perceived by psychiatrists as negativistic and the deconstruction as destructive.

In my opinion, neuroscience is currently much more successful in embracing philosophy than psychiatry. Philosophy interacts positively with neuroscience and the philosophy of neuroscience is accepted as a natural result. The emerging area of philosophy of neuroscience certainly was spurred by remarkable recent growth in the neurosciences. Cognitive neuroscience continues to encroach upon issues traditionally addressed within philosophy, including the nature of consciousness, action, knowledge, and morality. Examining the implications of neurological syndromes for the concept of a unified self as well as studying the neural systems underlying appraisal and its relevance to the self is one example [6]. Other examples (among many) include: The concept of neurophenomenology, introduced by Francesco Varela into neuroscience, in which observers examine their conscious experience using scientifically verifiable methods [7]. The use of deep brain electrical stimulation to modulate behavioral responsiveness in a patient who remains in a minimal conscious state (thereby offering a new tool to comprehend consciousness) [8]. Another topic examines threatened morality and physical cleansing, or the neural constituents of moral cognition [9]. Or the investigation of subjective certainty and its relationship to dopamine alterations in the striatum [10].

In closing, I agree that we live in interesting philosophical times in which there is potential for a fruitful crossover between the disciplines of philosophy and psychiatry. However, I disagree that there needs be a "new" cross-over between philosophy and psychiatry as regards values, meaning and facts. These three themes must necessarily be – as they have historically been – intrinsic to psychiatric thinking, as their "self evidence" has shaped psychiatry as a distinctive medical science. I would encourage the field of psychiatry to incorporate once again basic philosophical attitudes which render possible true dialogue with philosophy and consequently enrich both disciplines. At the moment, for most practicing psychiatrists, philosophy is a bridge too far.

The views that I develop here should not discredit the value and importance of Natalie Banner and Tim Thornton's paper and the excellent series "International Perspectives in Philosophy and Psychiatry." I hope that my comments that were inspired by their thoughtful paper may help emphasize the importance of philosophical reflection within psychiatry. As Jaspers said "Everybody inclined to disregard philosophy will be overwhelmed by philosophy in an unperceived way".

## Competing interests

The author(s) declare that they have no competing interests.
